# Microbiological Changes during Ripening of Chihuahua Cheese Manufactured with Raw Milk and Its Seasonal Variations

**DOI:** 10.3390/foods7090153

**Published:** 2018-09-17

**Authors:** Cristina Sánchez-Gamboa, Liliana Hicks-Pérez, Néstor Gutiérrez-Méndez, Norma Heredia, Santos García, Guadalupe Virginia Nevárez-Moorillón

**Affiliations:** 1 Facultad de Ciencias Biológicas, Universidad Autónoma de Nuevo León, Ave. Pedro de Alba s/n cruce con Ave. Manuel L. Barragán, 66450 San Nicolás de los Garza, Nuevo León, México; cris86_saga@hotmail.com (C.S.-G.); norma@microbiosymas.com (N.H.); santos@microbiosymas.com (S.G.); 2 Facultad de Ciencias Químicas, Universidad Autónoma de Chihuahua, Circuito Universitario s/n Campus Universitario II, 31125 Chihuahua, Chihuahua, México; lilyhicks_4@hotmail.com (L.H.-P.); ngutierrez@uach.mx (N.G.-M.)

**Keywords:** lactic acid bacteria, cheese ripening, foodborne indicators, Chihuahua cheese, microbial quality

## Abstract

Chihuahua cheese is a traditional cheese produced in Northwest Mexico that is consumed shortly after production. Cheeses prepared during autumn, winter and summer were collected from five dairies, and analyzed to determine seasonal influence on proximate analysis, texture profile and the microbiological dynamic during a ripening period of 270 days. Coliforms, coagulase-positive staphylococci, molds, yeast, as well as presumptive mesophilic lactobacilli, thermophilic lactobacilli, lactococci, thermophilic cocci and enterococci, were enumerated by plate count on selective agar. Manufacturing dairy had an effect on Chihuahua cheese composition and texture profile. Seasonality influence on the microbial dynamic was observed, since the highest initial counts of coliforms (5.14 log CFU/g), coagulase-positive staphylococci (4.13 log CFU/g) and mesophilic lactobacilli (7.86 log CFU/g) were detected on summer samples. Also, ripening time affected the survival of coliforms and presumptive lactococci after 270 days (1.24 and 5.89 log CFU/g respectively) while from day 90th, coagulase-positive staphylococci were absent. Microbial changes and seasonal influence provide information on the microbiota that can influence the sensorial characteristics of Chihuahua cheese.

## 1. Introduction

Cheese is the result of the metabolic action of the microorganisms already present or added to milk [1], and there are several factors that can impact on the microbial diversity of the product, including milking practices, livestock feed, environmental conditions [2,3], the season of the year in which the cheese is manufactured [4,5], as well as the dairy environment and the equipment used [6]. Once elaborated, some cheese varieties are ready to consume, while in others, a ripening process is necessary to allow the development of the particular characteristics of texture, aroma and flavor [7]. During ripening, biochemical reactions (proteolysis, lipolysis and glycolysis) associated with the particular flavor and texture of each cheese variety, are carried out by enzymes either produced by live microorganisms or released onto the cheese matrix after microbial cellular lysis [1]. Short ripening periods are observed for artisanal cheeses manufactured from raw milk, probably due to their large microbial diversity; such foods are characterized by marked and distinctive sensory attributes as compared with those prepared with pasteurized milk [3]. During ripening, the group of non-starter lactic acid bacteria (NSLAB) acts producing volatile aroma compounds from citrate, lipids, esters and amino acids [3,7].

Consumption of cheeses prepared with raw milk represents a health risk, due to the presence of pathogenic microorganisms; but during ripening, their presence or elimination is determined by such factors as moisture content, salt concentration and pH [2]. The competition for nutrients [6], the production of antimicrobial compounds such as bacteriocins [8,9], organic acids, ethanol, carbon dioxide, hydrogen peroxide, may create unfavorable conditions for the growth and survival of pathogenic microorganism in cheese [3,10].

In Mexico, Chihuahua cheese is the fifth type of cheese manufactured nationally, with an annual production in 2017 of 40,700 tons [11]; it is produced mainly in Chihuahua State, and its origin is closely related to the establishment of the Mennonite group in the northwest part if the State in 1922. This semi-hard, sliceable, pale-yellow cheese is produced from cow’s milk (raw or pasteurized) [12]; a large ripening period is not mandatory, and cheese is usually consumed within three to four months of manufacturing [13].

There are no reports on the microbiological changes that occur during aging of Chihuahua cheese, and the existing reports have been focused only on the study of physicochemical changes that occur in a maximum period of 16 weeks [13,14]. The artisanal product made with raw milk will disappear due to the mandatory heat treatment of the milk used for its elaboration; therefore, the study of the traditional product was important in order to describe its microbial changes throughout ripening. Differences in cheese processing and microbial quality can provide a baseline for improvement of product quality, while preserving its traditional characteristics.

## 2. Materials and Methods

### 2.1. Sample Collection

Blocks of Chihuahua cheese manufactured with raw milk without the addition of starter cultures or chemical additives were obtained from five artisanal dairies (A–E). Cheese manufacturing plants were visited in one occasion during autumn, winter and summer (2013–2014) and included two Mennonite farmers (B and C) and three dairies operated by non-Mennonite farmers (A, D and E). Cheeses were manufactured by a process that is based from the beginning to the end, on the experience of each producer [12]. Samples were taken after cheese pressing, and were transported in controlled refrigerated conditions to the laboratory, using their original packaging; thus, cheeses from factory A were transported vacuum packed, while cheeses manufactured on dairies B and C conserved their blanket wrap; cheeses collected from farms D and E were maintained only with a plastic cover. For each season, one block of cheese was obtained from the dairies, meaning that a total of 15 different cheeses were collected.

### 2.2. Texture and Proximate Analysis of Chihuahua Cheese

Chemical analysis of Chihuahua cheese was done using AOAC methods [15] for moisture (method 926.08), protein (method 991.22), total fat (method 933.05) and total ash (method 935.42), using cheese samples after one month of manufacturing. Texture profile analysis (TPA) was done in cheese samples after four months of ripening, considering that is usually the highest time of their consumption. Cheese samples were maintained under controlled conditions (6.5 °C, vacuum-packed) A 2 by 1-cm cylinder was obtained with a stainless-steel punches, and were placed in a closed container to avoid desiccation, until samples reached a 20 °C temperature. Samples were compressed up to 50% of their original size, using a 50 kg load using a crosshead speed of 2 mm/s, in TA.XTplus Texture Analyzer (Stable Micro Systems LTD, Godalming, UK) [16].

### 2.3. Cheese Ripenning and Sampling

Cheeses were analyzed 24–48 h after their elaboration (referred as time 0), once every 30 days during 6 months, and finally at 9 months of ripening (270 days). For aging, samples were vacuum packaged and maintained at 6.5 °C. Prior to microbial analysis, cheese surface was aseptically cleaned with ethanol 70% (*v/v*); the external layer of the cheese was discarded and a portion of 10 g was taken. The sample obtained was placed inside of a sterile plastic bag and 90 mL of sterile sodium phosphate buffer (PBS, pH 7.2) were added. A food homogenizer (BagMixer Interscience Model CC, Saint Nom, France) was used during 1 min to get a uniform mix, and was used to prepare decimal dilutions in PBS which were used to inoculate specific media for each microbial group.

### 2.4. Determination of Indicator Organisms

Enumeration of total coliforms was done using Violet Red Bile Agar (BD, Bioxon, México City, México) incubated on aerobic conditions during 24 h at 37 °C; molds and yeast were determined aerobically on Potato Dextrose Agar (BD, Bioxon, México City, México) acidified with 10% (*w*/*v*) (1.5 mL/100 mL) tartaric acid and incubated for 5 days at 25 °C. Presence of coagulase-positive staphylococci was determined spreading 100 µL of each dilution on Baird Parker Agar plates (BD, Bioxon, México City, México) enriched with egg yolk tellurite emulsion incubated at 37 °C for 2 days. Presumptive colonies were tested for catalase, coagulase and Gram strain, in order to identify them as coagulase positive staphylococci [12].

### 2.5. Lactic Acid Bacteria (LAB) Groups Enumeration

For the enumeration of LAB groups, plates of MRS (for determination of presumptive lactobacilli) and Elliker Agar (for determination of presumptive lactococci), were inoculated with 100 µL of the dilutions prepared from cheese sample (BD, Bioxon, México City, México). Plates were incubated on a reduced oxygen atmosphere (BD Gas Pack CO_2_, Becton Dickinson Co., Franklin Lakes, NJ, USA) for 2 days at 25 for mesophilic and 42 °C for thermophilic species. Also, presumptive enterococci were determined using Kanamycin Esculin Azide Agar, or Bile Esculin Agar (BD, Bioxon, México City, México) incubated on aerobic conditions during 2 days at 37 °C [12]. From the plates used for LAB enumeration, at least five distinctive colonies were further cultured to obtain pure cultures, and were tested for catalase and Gram stain for microscopic observation.

### 2.6. Statistical Analysis

Results of microbial counts are shown as the average value of triplicate microbial counts expressed as log CFU/g of sample; standard deviation was determined after log transformation. Results were subjected to analysis of variance, considering a repeated measurement design using ripening time as covariable. Independent variables used were cheese factory and sampling season; a Tukey’s test was used for mean comparison at the 5% significance level. Statistical analysis was done using the statistical software Minitab 17 [17].

## 3. Results and Discussion

### 3.1. Chihuahua Cheese Characterization and Texture Analysis

Results of proximate analysis of Chihuahua cheese are shown in Table 1, which also includes the parameters established in the Mexican Standard for Chihuahua cheese [18]; all cheese samples complied with the specifications of the standard, that is especially dedicated to assure that Chihuahua cheese is manufactured with whole bovine milk, without additives, in order to protect traditional cheese producers [12]. According to the statistical analysis, there was no significant difference in protein content among the dairies, but there were differences in fat content, ash and moisture. Differences in fat content can be attributed to characteristics of livestock feeding [19], while moisture in the final product is also related to final pressing time during cheese manufacturing; accordingly, dairy E had the shorter pressing time, of only 1 h. As previously reported, cheesemaking was different among dairies, which based their procedures on their experience; however, Chihuahua cheese quality can benefit from standardized procedures among producers [12].

Chihuahua cheese is considered a semi-matured cheese, with only a short period of ripening before being offered to the consumer (one month) [20]. It is usually consumed within three months, and its melt and stretch properties are considered important to define cheese quality. In order to evaluate the textural properties of the samples analyzed, cheese was maintained under controlled conditions for four months. Results are presented in Table 2 for each one of the dairies, considering that for most of the parameters tested, there was no significative differences between seasons. None of the samples presented fracturability, and there were no differences among dairies or season for hardness, cohesiveness and adhesiveness (*p* > 0.05). Regarding springiness, there were differences among seasons (winter, summer>autumn), but not between dairies (*p* > 0.05). The only parameter that presented highly significant difference among seasons (winter, summer > autumn) and dairies was chewiness (*p* < 0.01).

Cheese texture is the result of a complex interaction of factors such as cheese composition, manufacturing process and ripening conditions. Considering that cheese was manufactured with raw milk, conditions such as herd race, lactation time and quality of the animal feed, will influence cheese properties [21]. The rheological and textural properties of Chihuahua cheese manufactured with raw and pasteurized milk has been reported before, with differences on the type of cheese-milk used; results are similar to the ones reported here for 16 weeks of storage [22]. Another report on the texture properties of Chihuahua cheese manufactured with raw and pasteurized milk, also showed differences among sampling season and milk used; the analysis was done after 10 days of preparation, with higher values for chewiness that the ones reported here [23]. Lower values of chewiness after four months of ripening, can be explained by the proteolytic activity of non-starter LAB and the decrease in pH.

### 3.2. Ripening and Seasonal Effect on LAB Microbial Counts

Microbiological changes during aging promote the abundance and diversity of distinctive groups of microorganisms on the cheese matrix [24], whose metabolic activity is essential for the production of compounds related to particular sensorial characteristics [25]. On the other hand, the microbiological profile of any fermented dairy product such as cheese, is affected by the presence of native microorganisms and the milk chemical composition, which in turn is affected by seasonal variations of climatic conditions and animal feeding. Therefore, throughout the year it is possible to obtain cheeses of the same variety with differences on the microbial profile of different LAB groups, as well as variations in physicochemical and sensorial properties [4,19]. In the particular case of Mexican Chihuahua cheese, there are some reports of the effect of seasonality on rheological and textural properties [12,22,23], but there are no previous studies on the seasonal variations of microbial counts during its ripening.

Statistical analysis of LAB microbial counts considering all samples collected (15 cheese samples, from five dairy farmers collected throughout a year) showed that counts of all LAB groups were affected by the season in which the sample was obtained (*p* < 0.05). Changes in the LAB groups during the 270 days of analysis, are included in Supplemental Material (Figures S1–S5); the average of all dairies per season is shown in Table 3. The highest values of LAB were determined on cheeses produced during summer; the dominant microorganisms were *Lactobacillus* species. Based on statistical analysis, season of cheese production was the main factor that affected the dynamic of all LAB groups analyzed; a strong influence of ripening time (*p* < 0.05) was observed only in the cases of presumptive mesophilic lactobacilli, lactococci and thermophilic cocci.

Initial counts of presumptive mesophilic lactobacilli, lactococci and thermophilic cocci were 7.5, 6.8 and 6.4 log CFU/g respectively, and after 60 days of ripening, an increase was observed, to the decrease with mean values after the 270 days maturation period of 7.2 log CFU/g, for presumptive mesophilic lactobacilli, while presumptive lactococci numbers were 7.1 log CFU/g, and presumptive thermophilic cocci counts were 6.8 log CFU/g.

Results showed a clear predominance of lactobacilli during Chihuahua cheese maturation, and especially of presumptive mesophilic lactobacilli due to their highest numbers in comparison with the presumptive thermophilic lactobacilli group at the end of ripening. Therefore, presumptive mesophilic lactobacilli species could play a key role on maturation of Chihuahua cheese [26]. Another cheese varieties, such as Canestrato Pugliese [8], and Cueva de la Magahá cheese [24] have similar pattern of LAB microbial counts as Chihuahua cheese, with lactobacilli as the main microbial group during ripening. On the contrary, Fiore Sardo cheese showed a marked predominance of lactococci during early stages of ripening [27], while on a study of low-fat Cheddar cheese, *Lactococcus lactis* was present after 9 months of maturation [25].

In addition to ripening time and season in which cheese is manufactured, variation between dairy producers (*p* < 0.05) was observed in the case of thermophilic lactobacilli and presumptive enterococci, as can be observed in Table 4. Dairy influence on cheese microbial composition has been reported previously in Kaşar cheese [28].

Levels of presumptive enterococci were strongly influenced by season, the manufacturing dairy and maturation time (*p* < 0.05). The highest counts were observed on cheeses from dairies A and E (6.9 log CFU/g), while the lowest was of factory C (5.8 log CFU/g). The presence of this microorganism in cheese has been associated with the use of raw milk for cheese manufacturing [24], and their survival in ripped cheeses is related to their tolerance to high salt concentrations, low moisture environments, and low maturation temperatures [27]. Because of their proteolytic and lipolytic activity [2,29] and their citrate metabolism [29], enterococci excrete compounds such as acetaldehyde, acetoin and diacetyl on the cheese matrix, and have been related to cheese flavor development in traditional products [2]. Constant counts of enterococci were observed during maturation, which is in agreement with that reported for other cheese varieties such as Torta del Casar cheese [30], but differs from Cueva de la Magahá [24] and Fiore Sardo [27], where enterococci were in low numbers at the beginning of ripening and were undetectable after 9 months. While based on the statistical analysis, seasonality is an important factor that determine initial counts and/or the dominant LAB species, the presence of enterococci during aging can be related to the particular characteristics of each cheese variety, which in turns is the result of the manufacturing practices of each factory, type and characteristics of milk used, storage conditions and physicochemical changes that may occur during ripening [4,19].

Variations within the same dairy at the three different sampling times were also observed, such as in farm C, where cheese manufactured in summer had the highest levels of presumptive mesophilic lactobacilli, thermophilic cocci and enterococci (8.4, 8.1 and 7.7 log CFU/g, respectively) at early stages of ripening; but the cheese produced in autumn, had the lowest counts of presumptive mesophilic and thermophilic lactobacilli as well as lactococci counts (6.4, 5.9 and 5.3 log CFU/g, respectively) of all dairies tested. On the contrary, there is no explanation of why cheeses from different dairies (located on different geographical regions) had similar pattern of some microorganisms, as was the case of cheeses A and C from autumn that had undetectable counts of presumptive lactococci and thermophilic cocci after 6 months of maturation, and the lowest levels of presumptive mesophilic lactobacilli at the end of the study.

### 3.3. Influence of Ripening Time and Season on Microbial Counts of Indicator Microorganisms

Raw milk from a healthy udder should be sterile, but microorganisms from different sources are incorporated into milk [2,3,31]. Certainly, fermenting microorganisms such as LAB, can reduce the growth of pathogenic and spoilage bacteria, either by microbial competition for milk nutrients or by producing acid that diminishes the pH of the food product [31]. Due to the use of raw milk for the manufacture of the Chihuahua cheese samples analyzed, high counts of indicator organisms were detected, especially on samples taken during summer. In fact, statistical analysis for indicator microorganisms from the five dairies visited, indicated that microbial counts were the most important factor that affects their microbial counts is the season in which the cheese was manufactured (*p* < 0.05), as shown in Table 3.

Elevated counts of coliforms on freshly prepared cheeses (time 0) were observed on summer samples, whose media of counts was 6.9 log CFU/g; followed by the ones sampled during winter (6.4 log CFU/g) and autumn (5.9 log CFU/g). From all the 15 samples, cheese B obtained during summer sampling had the highest count of coliforms (7.6 log CFU/g), while the lowest was cheese C manufactured during autumn (5.2 log CFU/g). Although coliform bacteria are common members of microbial community of cheeses made from raw milk, their presence in cheese is mostly associated with deficient hygienic practices during milking and manufacturing [10,30], unrefrigerated storage, transportation [10], and also to a low acid production during milk fermentation [5].

**Figure 1:**
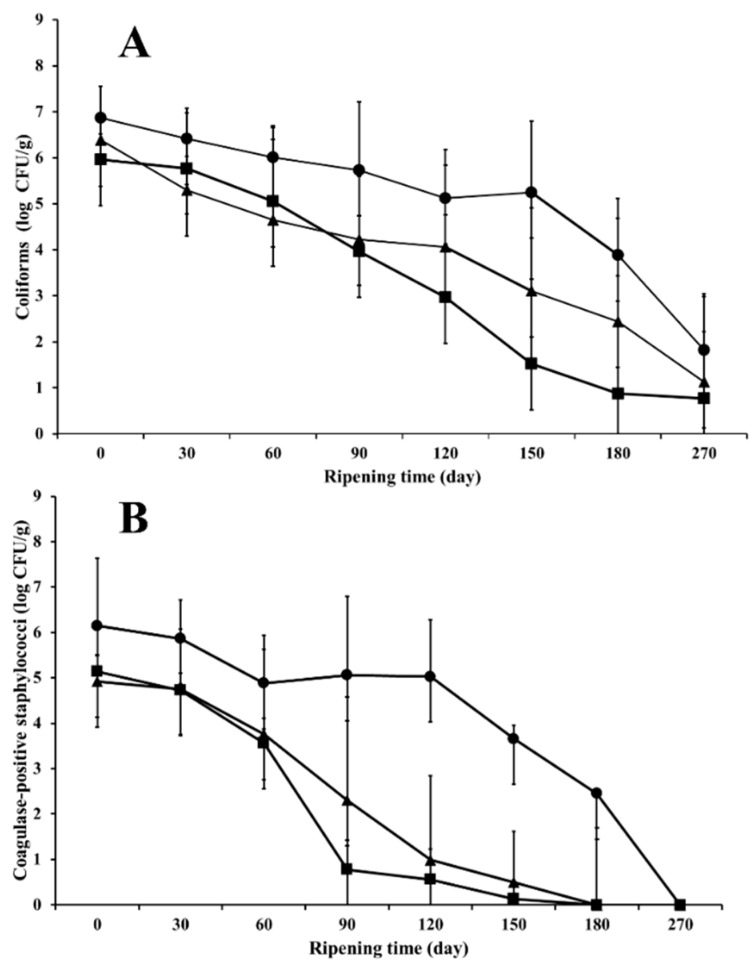
Microbial dynamic during ripening of Chihuahua cheese manufactured with raw milk during autumn (■), winter (▲) and summer (●): (**A**) coliforms; (**B**) coagulase-positive staphylococci counts.

There is a strong influence of ripening time and dairy plant (*p* < 0.05), on microbial count of total coliforms. In Figure 1A, the evolution of coliforms during ripening throughout the year can be observed; while Table 4 shows the influence of the dairy sampled. After 150 days of maturation, coliforms were undetectable on cheeses manufactured on farm E during autumn and winter; on day 180th, coliforms were absent on autumn cheese C and winter cheese B. In contrast, even afterwards 270 days of aging, slightly higher coliform counts were observed in some samples: cheeses A and D from summer (3.2 and 5.1 log CFU/g, respectively), cheese A from autumn (2.6 log CFU/g) and cheese C from winter (2.4 log CFU/g). Similar pattern of coliform has been reported previously for other cheese varieties, such as Canestrato Pugliese [8] and Fiore Sardo [10,27], where coliforms were undetectable at the end of ripening period analyzed. The decrease or absence of coliforms during cheese ripening, has been attributed to a decrease in pH as a consequence of the fermentative activity of LAB as well as their antimicrobial compounds (bacteriocins, organic acids, ethanol, H_2_O_2_), in addition to the physicochemical changes produced in the cheese matrix [10,27].

Highest levels of coagulase-positive staphylococci were noticed on all cheese samples at the beginning of the ripening stage; cheese samples from dairies D and E manufactured during autumn registered the highest counts of this microbial group (6.6 and 6.8 log CFU/g, respectively), as well as cheese manufactured during summer on farm E (6.6 log CFU/g). Like coliform, coagulase-positive staphylococci levels were drastically affected by the maturation time and were also related to the dairy plant where the cheese was manufactured (*p* < 0.05). After 60 days of maturation, coagulase-positive staphylococci were undetectable in cheese C from winter; and after 90 days on autumn cheeses A, B, C and D, and in winter cheese B. The trend of coagulase-positive staphylococci counts over time can be observed in Figure 1B.

Among the coagulase-positive staphylococci, *S. aureus* in cheese and other foodstuff is of public health concern, because of the ability of some strains to produce water-soluble, heat-resistant enterotoxins [32]; this enterotoxin can be accumulated and cause food intoxication when 5–10 log CFU/g are found in the foodstuff [33]. In Mozzarella cheese, this Gram positive cocci can survive during a maturation period of 41 days [34]; presence of bacteriocin-producing LAB strains can be related to the decrease of *S. aureus* on cheese, as reported by Arqués et al. [35]. According to other reports, *S. aureus* was not detected in Fiore Sardo cheese after 1 month of ripening [27], or detected only at the end of ripening period (34 weeks) in products such as in Cueva de la Magahá cheese [24].

The population of yeast and molds increased during the first 60 days of ripening, time at which they reached their maximum level (5.8 and 4.3 log CFU/g, respectively); however, while counts of molds tended to decrease after that time, yeasts counts slightly decreased and remained constant during the rest of the maturation period. Strong influence of factory and ripening time was observed on counts of molds (*p* < 0.05), but season had no effect (*p* = 0.09). On freshly prepared cheeses, the highest counts of molds were observed on samples from cheese C prepared during autumn (5.9 log CFU/g), while the lowest were obtained from winter cheese E (1.6 log CFU/g). At the end of the ripening period analyzed, molds were undetectable on cheeses A, D and E from the autumn, but in cheese B from winter, counts were high (5.0 log CFU/g). Similar profile of molds during ripening was observed in Canestrato Pugliese cheese [8].

The counts of yeast were affected by season (*p* < 0.05) but not by the ripening time (*p* = 0.09). Initial yeast counts from cheese C manufactured during autumn had the highest counts (6.7 log CFU/g), while cheese D from winter had the lowest (3.9 log CFU/g). After 270 days, elevated counts in cheese E from winter were observed (6.4 log CFU/g), while they were absent in cheese from dairy A manufactured in summer. In other cheese varieties, such as Canestrato Pugliese, a similar behavior on the dynamics of yeast and molds was reported [8]; however, although our results suggest that microbial counts of yeast and molds are strongly influenced by the season of the year in which the cheese is elaborated, Cardoso et al. [36] found that in the case of Serro Minas Cheese seasonality did not exert significant influence on yeast counts. In addition to their lipolytic and proteolytic activities, yeast promote the growth of bacteria sensitive to low pH, by the increase of pH owing to their ability to metabolize lactic acid during ripening [37]. Although proteolytic and lipolytic activity of molds may contribute to some alterations of texture and aroma in cheese [2], their presence have not been widely studied. The differences on yeast and mold counts between dairies during the maturation period studied, can indicate their dependence on cheese physicochemical changes during ripening, which can be influenced by manufacturing practices and/or environmental conditions of each dairy location.

## 4. Conclusions

Chihuahua cheese manufactured with raw milk in different dairies located in the state of Chihuahua, Mexico, showed differences in proximate analysis depending on the manufacturing location, and their texture profile after four months of ripening was different only for chewiness. Seasonality was an important factor to determine the initial counts of indicators and LAB microbial groups. *Lactobacillus* were predominant during ripening and their metabolic activity could be determinant on sensorial and rheological changes during cheese maturation. Due to their artisanal origin, the distinctive characteristics of Chihuahua cheese are the result of numerous microbial interactions and microbial activity, which is not at all exclusive of LAB groups, since other microorganism could contribute to the development of cheese attributes (aroma, texture, and flavor). Further investigation is necessary in order to identify strains with high potential to be used in the elaboration of Chihuahua cheese from pasteurized milk.

## Figures and Tables

**Table 1:** Proximate analysis of Chihuahua cheese manufactured with raw milk in five different dairies.

Cheese Factory	Moisture (%)	Fat (%)	Protein (%)	Ash (%)
A	39.3 ± 1.5 ^ab^	22.2 ± 1.7 ^c^	27.2 ± 0.7 ^a^	4.2 ± 0.2 ^ab^
B	39.2 ± 1.2 ^ab^	40.1 ± 2.5 ^a^	25.6 ± 2.6 ^a^	3.7 ± 0.3 ^b^
C	37.6 ± 1.0 ^ab^	32.8 ± 2.6 ^b^	29.9 ± 2.5 ^a^	3.6 ± 0.2 ^b^
D	35.5 ± 1.3 ^b^	34.0 ± 2.1 ^b^	26.9 ± 0.9 ^a^	4.9 ± 0.4 ^a^
E	41.6 ± 3.0 ^a^	30.3 ± 1.6 ^b^	27.0 ± 0.7 ^a^	5.0 ± 0.3 ^a^
Mexican Standard [18]	Max 45	Min 28	Min 23	

Data are expressed as mean values ± standard deviation for each parameter considering cheese samples during the three sampling periods, each determination was done in triplicate. Means with different letter within each column are significantly different (*p* < 0.05) according to Tukey mean analysis.

**Table 2:** Texture profile analysis (TPA) analysis of Chihuahua cheese manufactured with raw milk in five different dairies, after four months of ripening.

Cheese Factory	Hardness (N)	Adhesiveness (N s^−2^)	Springiness (mm)	Cohesiveness	Chewiness (mJ)
A	19.9 ± 9.0 ^a^	−0.80 ± 0.29 ^a^	9.5 ± 2.8 ^a^	0.33 ± 0.07 ^a^	77.3 ± 45.3 ^a^
B	15.7 ± 7.6 ^a^	−0.40 ± 0.26 ^a^	11.4 ± 4.3 ^a^	0.54 ± 0.29 ^a^	53.9 ± 27.5 ^a^
C	18.6 ± 4.6 ^a^	−1.1 ± 0.09 ^a^	11.0 ± 4.1 ^a^	0.53 ± 0.27 ^a^	82.4 ± 48.9 ^b^
D	26.3 ± 7.4 ^a^	−0.40 ± 0.46 ^a^	11.1 ± 2.4 ^a^	0.60 ± 0.22 ^a^	150.6 ± 44.7 ^a^
E	24.7 ± 12.0 ^a^	−0.91 ± 0.76 ^a^	10.9 ± 2.9 ^a^	0.41 ± 0.14 ^a^	102.3 ± 25.0 ^ab^

Data are expressed as mean values ± standard deviation for each parameter considering cheese samples during the three sampling periods, each measure is the average of ten determinations in a sample. Means with different letter within each column are significantly different (*p* < 0.05) according to Tukey mean analysis.

**Table 3:** Seasonal influence on microbial counts (log CFU/g) during aging of Chihuahua cheese manufactured with raw milk.

Microbial Group/Season	Autumn	Winter	Summer
Coliforms	3.3 ± 2.3 ^b^	3.9 ± 2.1 ^b^	5.1 ± 2.2 ^a^
Mold	3.1 ±1.6 ^ab^	2.7 ± 1.7 ^b^	3.6 ± 1.7 ^a^
Yeast	4.7 ± 1.2 ^a^	5.6 ± 1.3 ^a^	5.5 ± 1.6 ^a^
Presumptive staphylococci	1.9 ± 1.9 ^b^	2.2 ± 2.2 ^b^	4.1 ± 2.0 ^a^
Mesophilic lactobacilli	7.3 ±1.4 ^b^	7.7 ± 1.6 ^a^	7.9 ± 1.2 ^a^
Thermophilic lactobacilli	7.0 ± 1.3 ^b^	6.9 ± 0.6 ^b^	7.5 ± 0.5 ^a^
Presumptive lactococci.	5.6 ± 2.3 ^b^	6.9 ± 1.4 ^a^	7.1 ± 1.6 ^a^
Thermophilic cocci	5.0 ± 2.6 ^c^	6.0 ± 1.2 ^b^	6.8 ± 1. 5 ^a^
Presumptive enterococci.	6.4 ± 1.5 ^b^	5.8 ± 1.2 ^c^	7.5 ± 0.5 ^a^

Microbial counts on each season are expressed as mean values ± standard deviation for each microorganism obtained from counts of five cheeses during a period of 270 days of ripening. Means with different letter within each microbial group are significantly different (*p* < 0.05) according to Tukey mean analysis.

**Table 4:** Microbial counts (log CFU/g) observed during ripening of Chihuahua cheese manufactured with raw milk in five different dairies.

Microbial Group/Cheese Factory	A	B	C	D	E
Coliforms	4.6 ±1.9 ^ab^	4.2 ±2.5 ^b^	4.0 ± 2.0 ^b^	5.3 ± 1.9 ^a^	2.6 ± 2.4 ^c^
Mold	2.2 ± 1.6 ^c^	3.1 ± 1.6 ^bc^	4.3 ± 1.2 ^a^	2.8 ± 1.8 ^bc^	3.4 ± 1.5 ^ab^
Yeast	5.0 ± 1.5 ^a^	5.0 ± 1.1 ^a^	5.4 ± 0.9 ^a^	5.4 ± 1.2 ^a^	5.5 ± 1.2 ^a^
*S. aureus*	2.6 ±2.4 ^b^	2.6 ± 2.6 ^b^	2.1 ± 2.4 ^b^	2.6 ± 2.4 ^b^	3.7 ± 2.3 ^a^
Mesophilic lactobacilli	7.6 ± 1.0 ^a^	7.7 ±0.6 ^a^	7.4 ± 0.7 ^a^	7.7 ± 0.4 ^a^	7.7 ± 0.4 ^a^
Thermophilic lactobacilli	7.0 ± 0.6 ^b^	7.2 ± 0.7 ^ab^	6.8 ± 0.8 ^b^	7.6 ± 0.5 ^a^	7.2 ± 0.6 ^ab^
*Lactococcus* sp.	6.4 ± 2.1 ^a^	6.5 ± 1.2 ^a^	6.0 ± 2.1 ^a^	7.1 ± 0.5 ^a^	6.7 ± 0.8 ^a^
Thermophilic cocci	5.5 ± 2.3 ^a^	5.8 ± 2.0 ^a^	5.8 ± 2.0 ^a^	6.4 ± 1.1 ^a^	6.3 ± 0.6 ^a^
*Enterococcus* sp.	6.9 ± 0.7 ^a^	6.3 ± 1.0 ^bc^	5.8 ± 1.5 ^c^	6.8 ± 1.1 ^ab^	6.9 ± 0.8 ^a^

Microbial counts are expressed as mean values ± standard deviation for each microorganism obtained in during maturation of the three cheeses collected in each different dairy. Means with different letter within each microbial group are significantly different (*p* < 0.05) according to Tukey mean analysis.

## Supplementary Materials

The following are available online at http://www.mdpi.com/2304-8158/7/9/153/s1, Figure S1: Microbial dynamic of presumptive mesophilic lactobacilli during ripening of Chihuahua cheese manufactured with raw milk during autumn (■), winter (▲) and summer (●). Data is the average and standard deviation of all five dairies included in the study, Figure S2: Microbial dynamic of presumptive thermophilic lactobacilli during ripening of Chihuahua cheese manufactured with raw milk during autumn (■), winter (▲) and summer (●). Data is the average and standard deviation of all five dairies included in the study, Figure S3: Microbial dynamic of presumptive Lactococcus sp. during ripening of Chihuahua cheese manufactured with raw milk during autumn (■), winter (▲) and summer (●). Data is the average and standard deviation of all five dairies included in the study, Figure S4: Microbial dynamic of presumptive thermophilic cocci during ripening of Chihuahua cheese manufactured with raw milk during autumn (■), winter (▲) and summer (●). Data is the average and standard deviation of all five dairies included in the study, Figure S5: Microbial dynamic of presumptive Enterococcus during ripening of Chihuahua cheese manufactured with raw milk during autumn (■), winter (▲) and summer (●). Data is the average and standard deviation of all five dairies included in the study.

## References

[1] Bautista-Gallego J., Alessandria V., Fontana M., Bisotti S., Taricco S., Dolci P., Cocolin L., Rantsiou K. (2014). Diversity and functional characterization of *Lactobacillus* spp. isolated throughout the ripening of a hard cheese. Int. J. Food Microbiol..

[2] Quigley L., O’sullivan O., Stanton C., Beresford T.P., Ross R.P., Fitzgerald G.F., Cotter P.D. (2013). The complex microbiota of raw milk. FEMS Microbiol. Rev..

[3] Montel M.C., Buchin S., Mallet A., Delbes-Paus C., Vuitton D.A., Desmasures N., Berthier F. (2014). Traditional cheeses: Rich and diverse microbiota with associated benefits. Int. J. Food Microbiol..

[4] Fernández-García E., Gaya P., Medina M., Nuñez M. (2004). Evolution of the volatile components of raw ewes’ milk Castellano cheese: Seasonal variation. Int. Dairy J..

[5] Wullschleger S., Lacroix C., Bonfoh B., Sissoko-Thiam A., Hugenschmidt S., Romanens E., Baumgartner S., Traoré I., Yaffee M., Jans C. (2013). Analysis of lactic acid bacteria communities and their seasonal variations in a spontaneously fermented dairy product (Malian fènè) by applying a cultivation/genotype-based binary model. Int. Dairy J..

[6] Scatassa M.L., Gaglio R., Macaluso G., Francesca N., Randazzo W., Cardamone C., Di Grigoli A., Moschetti G., Settanni L. (2015). Transfer, composition and technological characterization of the lactic acid bacterial populations of the wooden vats used to produce traditional stretched cheeses. Food Microbiol..

[7] Broadbent J.R., Steele J.L. (2005). Cheese flavor and the genomics of lactic acid bacteria. ASM NEWS.

[8] De Pasquale I., Calasso M., Mancini L., Ercolini D., La Storia A., De Angelis M., Di Cargo R., Gobbetti M. (2014). Causal relationship between microbial ecology dynamics and proteolysis during manufacture and ripening of protected designation of origin (PDO) cheese Canestrato Pugliese. Appl. Environ. Microb..

[9] Pogačić T., Mancini A., Santarelli M., Bottari B., Lazzi C., Neviani E., Gatti M. (2013). Diversity and dynamic of lactic acid bacteria strains during aging of a long ripened hard cheese produced from raw milk and undefined natural starter. Food Microbiol..

[10] Piras C., Marincola F.C., Savorani F., Engelsen S.B., Cosentino S., Viale S., Pisano M.B. (2013). A NMR metabolomics study of the ripening process of the Fiore Sardo cheese produced with autochthonous adjunct cultures. Food Chem..

[11] Secretaría de Agricultura, Ganadería, Desarrollo Rural, Pesca y Alimentación (SAGARPA), Servicio de Información Agroalimentaria y Pesquera (SIAP) Boletín de leche. Enero-Marzo 2018. http://infosiap.siap.gob.mx/opt/boletlech/Bolet%C3%ADn%20de%20Leche%20enero-marzo%202018.pdf.

[12] Sánchez-Gamboa C., Hicks-Pérez L., Gutiérrez-Méndez N., Heredia N., García S., Nevárez-Moorillón G.V. (2018). Seasonal influence on the microbial profile of Chihuahua cheese manufactured from raw milk. Int. J. Dairy Technol..

[13] Olson D.W., Van Hekken D.L., Tunick M.H., Tomasula P.M., Molina-Corral F.J., Gardea A.A. (2011). Mexican Queso Chihuahua: Functional properties of aging cheese. J. Dairy Sci..

[14] Paul M., Nuñez A., Van Hekken D.L., Renye J.A. (2014). Sensory and protein profiles of Mexican Chihuahua cheese. J. Food Sci. Technol..

[15] AOAC (1998). Official Methods of Analysis of AOAC International.

[16] Brennan J.G., King R.D. (1980). Food texture measurement, In Developments in Food Analysis Techniques.

[17] (2016). Minitab 17 Statistical Software [Computer software].

[18] Diario Oficial de la Federación (DOF) (2011). MNX-F-738-COFOCALEC-2011. Sistema Producto Leche-Lácteos-Queso Chihuahua-Denominación, Especificaciones y Métodos de Prueba. http://www.cofocalec.org.mx/catalogo/por_clave.

[19] Broadbent J.R., Brighton C., McMahon D.J., Farkye N.Y., Johnson M.E., Steele J.L. (2013). Microbiology of Cheddar cheese made with different fat contents using a *Lactococcus lactis* single-strain starter. J. Dairy Sci..

[20] Diario Oficial de la Federación (DOF) Norma Oficial Mexicana NOM-243-SSA1-2010. Productos y Servicios. Leche, Formula Láctea, Producto Lácteo Combinado y Derivados Lácteos. Disposiciones y Especificaciones Sanitarias. Métodos de Prueba. Diciembre 2012. http://dof.gob.mx/nota_detalle.php?codigo=5160755&fecha=27/09/2010.

[21] Lucey J.A., Johnson M.E., Horne D.S. (2003). Perspectives on the basis of the rheology and texture properties of cheese. J. Dairy Sci..

[22] Tunick M.H., Van Hekken D.L., Call J., Molina-Corral F.J., Gardea A.A. (2007). Queso Chihuahua: Effects of seasonality of cheesemilk on rheology. Int. J. Dairy Technol..

[23] Van Hekken D.L., Drake M.A., Tunick M.H., Guerrero V.M., Molina-Corral F.J., Gardea A.A. (2008). Effect of pasteurization and season on the sensorial and rheological traits of Mexican Chihuahua cheese. Dairy Sci. Technol..

[24] Martín-Platero A.M., Valdivia E., Maqueda M., Martín-Sánchez I., Martínez-Bueno M. (2008). Polyphasic approach to bacterial dynamics during the ripening of Spanish farmhouse cheese, using culture-dependent and-independent methods. Appl. Environ. Microb..

[25] Addis M., Fiori M., Riu G., Pes M., Salvatore E., Pirisi A. (2015). Physico-chemical characteristics and acidic profile of PDO Pecorino Romano cheese: Seasonal variation. Small Rumin. Res..

[26] Wouters J.T., Ayad E.H., Hugenholtz J., Smit G. (2002). Microbes from raw milk for fermented dairy products. Int. Dairy J..

[27] Pisano M.B., Fadda M.E., Deplano M., Corda A., Cosentino S. (2006). Microbiological and chemical characterization of Fiore Sardo, a traditional Sardinian cheese made from ewe’s milk. Int. J. Dairy Technol..

[28] Aydemir O., Harth H., Weckx S., Dervişoğlu M., De Vuyst L. (2015). Microbial communities involved in Kaşar cheese ripening. Food Microbiol..

[29] Martín-Platero A.M., Maqueda M., Valdivia E., Purswani J., Martínez-Bueno M. (2009). Polyphasic study of microbial communities of two Spanish farmhouse goats’ milk cheeses from Sierra de Aracena. Food Microbiol..

[30] Ordiales E., Martín A., Benito M.J., Hernández A., Ruiz-Moyano S., de Guía Córdoba M. (2013). Role of the microbial population on the flavor of the soft-bodied cheese Torta del Casar. J. Dairy Sci..

[31] Verraes C., Vlaemynck G., Van Weyenberg S., De Zutter L., Daube G., Sindic M., Uyttendaele M., Herman L. (2015). A review of the microbiological hazards of dairy products made from raw milk. Int. Dairy J..

[32] Schelin J., Wallin-Carlquist N., Thorup Cohn M., Lindqvist R., Barker G.C. (2011). The formation of *Staphylococcus aureus* enterotoxin in food environments and advances in risk assessment. Virulence.

[33] O’Brien M., Hunt K., McSweeney S., Jordan K. (2009). Occurrence of foodborne pathogens in Irish farmhouse cheese. Food Microbiol..

[34] Morea M., Baruzzi F., Cocconcelli P.S. (1999). Molecular and physiological characterization of dominant bacterial populations in traditional Mozzarella cheese processing. J. Appl. Microbiol..

[35] Arqués J.L., Rodríguez E., Gaya P., Medina M., Guamis B., Nunez M. (2005). Inactivation of *Staphylococcus aureus* in raw milk cheese by combinations of high-pressure treatments and bacteriocin-producing lactic acid bacteria. J. Appl. Microbiol..

[36] Cardoso V.M., Borelli B.M., Lara C.A., Soares M.A., Pataro C., Bodevan E.C., Rosa C.A. (2015). The influence of seasons and ripening time on yeast communities of a traditional Brazilian cheese. Food Res. Int..

[37] Mounier J., Monnet C., Vallaeys T., Arditi R., Sarthou A.S., Hélias A., Irlinger F. (2008). Microbial interactions within a cheese microbial community. Appl. Environ. Microb..

