# Supplementary material for: Microbiological Changes during Ripening of Chihuahua Cheese Manufactured with Raw Milk and Its Seasonal Variations
Source: Foods. 2018 Sep 17;7(9):153. doi: 10.3390/foods7090153 (PMC6163408; doi:10.3390/foods7090153)
Supplement: Supplementary file 1 [file foods-07-00153-s001.pdf]

SUPPLEMENTARY MATERIAL

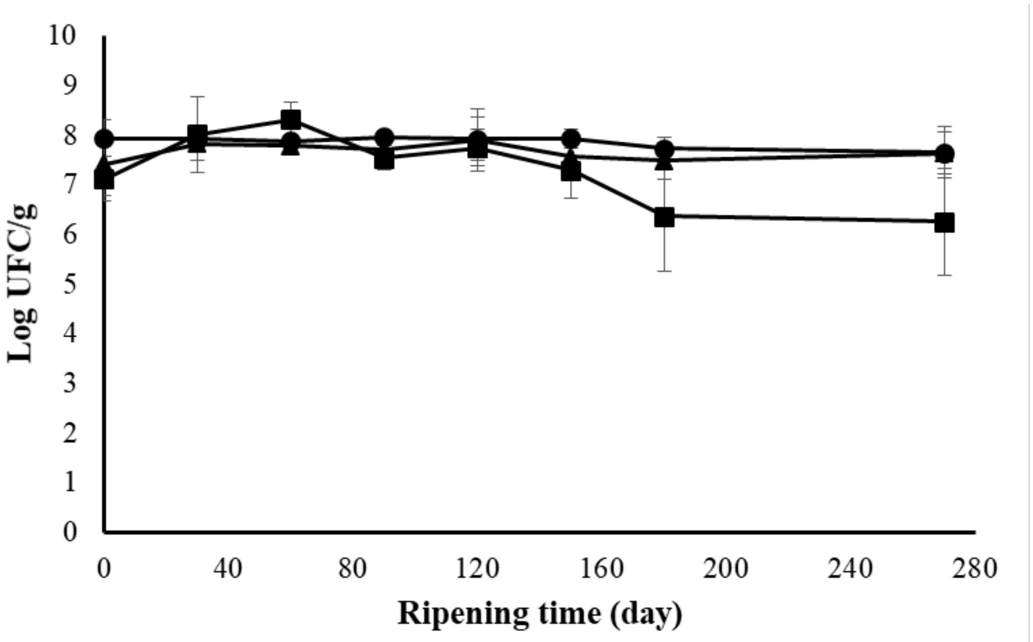

Figure S1. Microbial dynamic of presumptive mesophilic lactobacilli during ripening of Chihuahua cheese manufactured with raw milk during autumn (■), winter (▲) and summer (●). Data is the average and standard deviation of all five dairies included in the study.

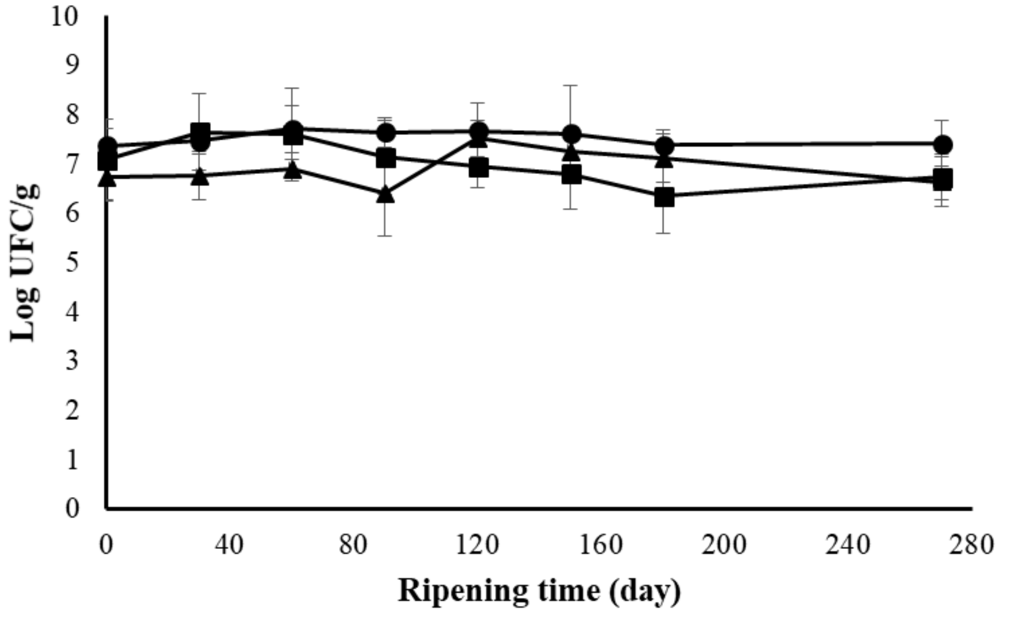

Figure S2. Microbial dynamic of presumptive thermophilic lactobacilli during ripening of Chihuahua cheese manufactured with raw milk during autumn (■), winter (▲) and summer (●). Data is the average and standard deviation of all five dairies included in the study.

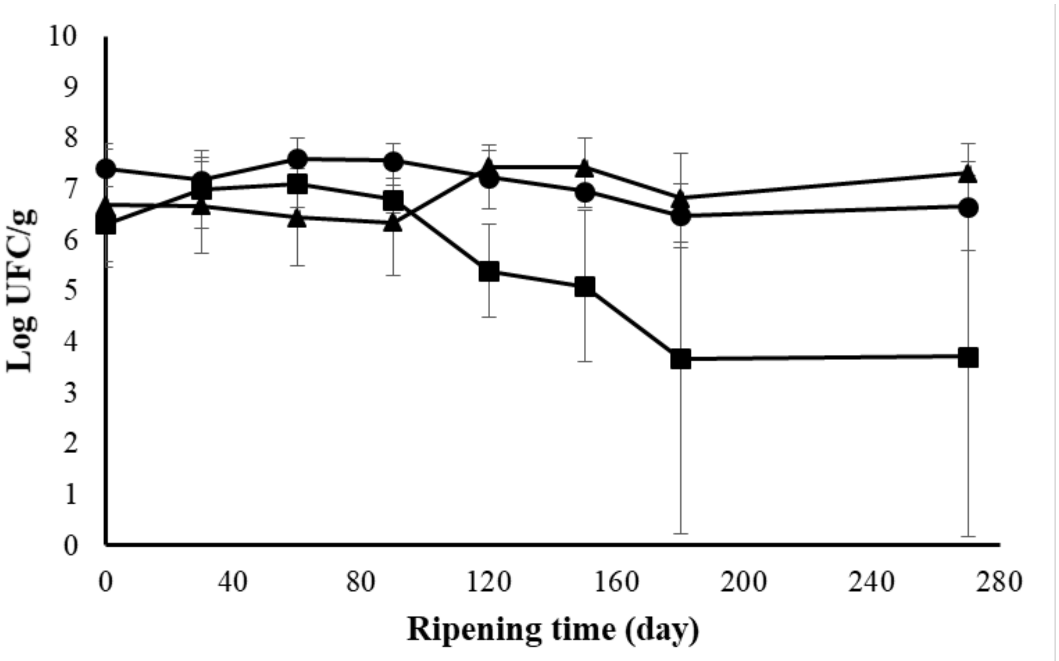

Figure S3. Microbial dynamic of presumptive *Lactococcus* sp. during ripening of Chihuahua cheese manufactured with raw milk during autumn (■), winter (▲) and summer (●). Data is the average and standard deviation of all five dairies included in the study.

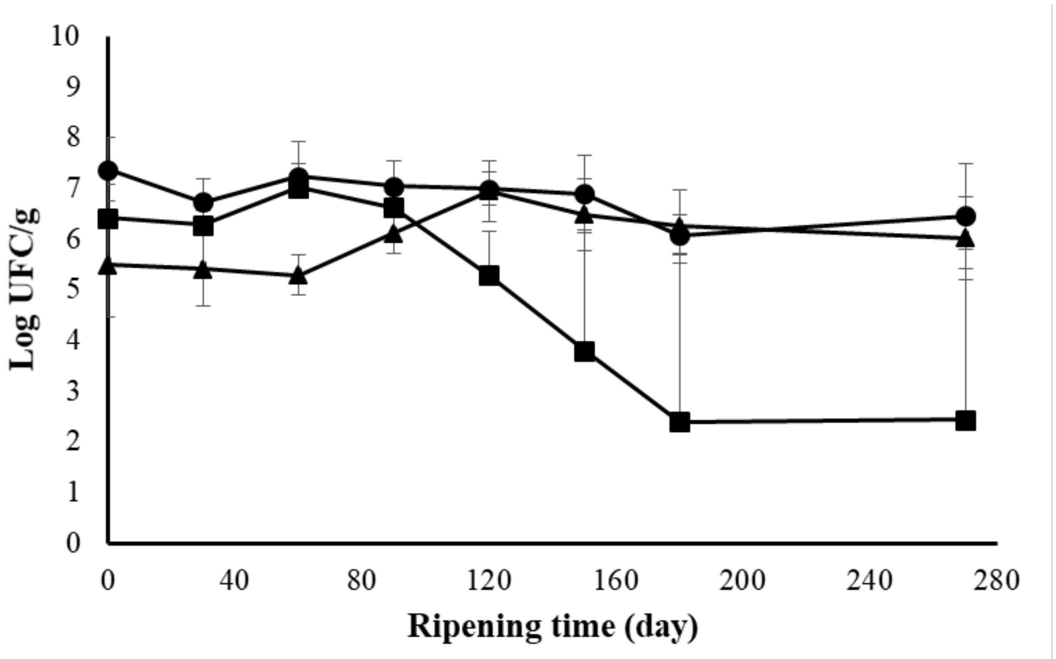

Figure S4. Microbial dynamic of presumptive thermophilic cocci during ripening of Chihuahua cheese manufactured with raw milk during autumn (■), winter (▲) and summer (●). Data is the average and standard deviation of all five dairies included in the study.

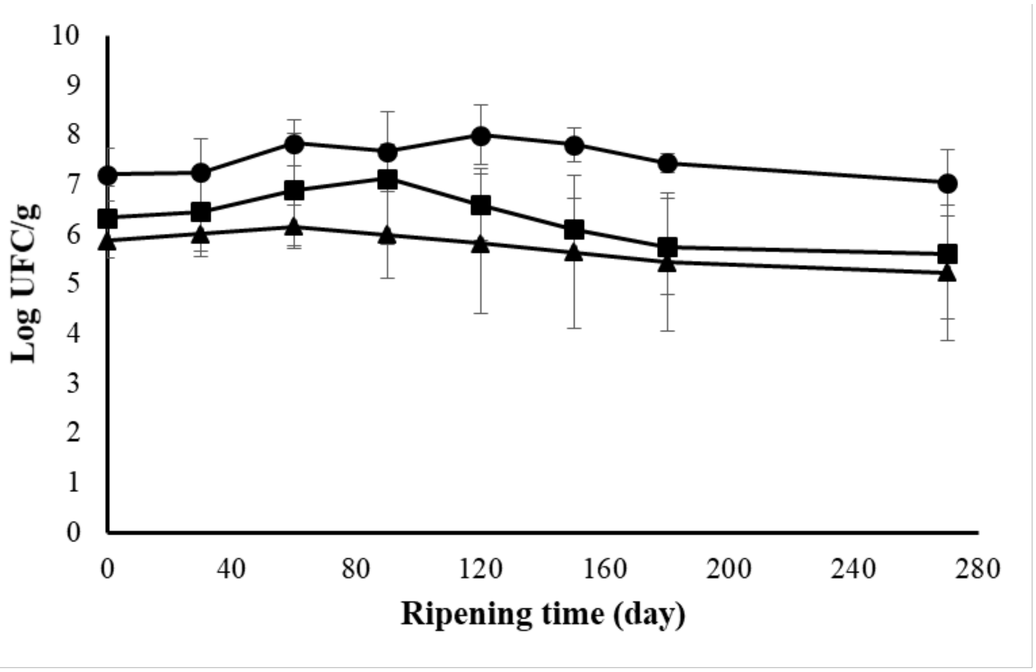

Figure S5. Microbial dynamic of presumptive Enterococcus during ripening of Chihuahua cheese manufactured with raw milk during autumn (■), winter (▲) and summer (●). Data is the average and standard deviation of all five dairies included in the study.
